# Preparation of p-type Fe_2_O_3_ nanoarray and its performance as photocathode for photoelectrochemical water splitting

**DOI:** 10.3389/fchem.2025.1526745

**Published:** 2025-01-24

**Authors:** Xiaoli Fan, Fei Zhu, Zeyi Wang, Xi Wang, Yi Zou, Bin Gao, Li Song, Jianping He, Tao Wang

**Affiliations:** ^1^ Jiangsu Key Laboratory of Advanced Structural Materials and Application Technology, School of Materials Science and Engineering, Nanjing Institute of Technology, Nanjing, China; ^2^ College of Materials Science and Technology, Nanjing University of Aeronautics and Astronautics, Nanjing, China; ^3^ School of Environmental Science and Engineering, Nanjing University of Information Science & Technology, Nanjing, China

**Keywords:** iron oxide, p-type semiconductor, photoelectrochemical water splitting, band structure, nanoarray structure

## Abstract

Photoelectrochemical (PEC) water splitting has the potential to convert solar energy into chemical energy, emerging as a promising alternative to fossil fuel combustion. In PEC systems, p-type semiconductors are particularly noteworthy for their ability to directly produce hydrogen. In this work, Fe_2_O_3_ with p-type semiconductor properties grown directly on the conductive glass substrate were successfully synthesized through a simple one-step hydrothermal method. The analysis results indicate that the Fe_2_O_3_ exhibits a spindle shaped nanoarray structure and possesses a small band gap, thereby demonstrating excellent photoelectrochemical performance as a photocathode with photocurrent density of −23 μA cm^−2^ at 0.4 V vs. RHE. Further band structure tests reveal that its conduction band position is more negative compared to the hydrogen evolution potential, highlighting its significant potential as a photocathode material.

## 1 Introduction

The main challenges facing human society stem from environmental pollution and the energy crisis, necessitating the exploration of green and sustainable alternative energies. Photoelectrochemical (PEC) water splitting, which converts solar energy into reusable chemical energy through semiconductors, offers a viable path for replacing fossil fuels (Chen et al., 2010; Daulbayev et al., 2020; Hansora et al., 2024). Currently, the majority of research on PEC water splitting focuses on n-type semiconductors, such as binary oxides like TiO_2_ (Liu et al., 2023; Wang et al., 2021), ZnO (Hieu et al., 2020; Xiao et al., 2017), α-Fe_2_O_3_ (Wang C. et al., 2019; Luo et al., 2017), WO_3_ (Shao et al., 2020), and ternary oxides like BiVO_4_ (Gao et al., 2020; Gao et al., 2022), Bi_2_WO_6_ (Dong et al., 2017), CuWO_4_ (Li and Diao, 2020), which are used as photoanodes in PEC systems. These materials are favored due to their abundance, non-toxicity, stability, and ease of low-cost preparation. However, p-type semiconductors are particularly noteworthy for their ability to directly produce hydrogen when used as photocathodes (Li et al., 2023). Therefore, the search for efficient p-type semiconductors with appropriate bandgap structures is a challenging yet significant task.

Currently reported p-type semiconductors include Si-based semiconductors (Sun et al., 2014; Hu et al., 2014), III-V group semiconductors [e.g., InP (Lin et al., 2015), InGaN (Alotaibi et al., 2013)], Cu-based oxide semiconductors [e.g., Cu_2_O (Li et al., 2019), CuFeO_2_ (Read et al., 2012)], and Fe-based oxide semiconductors (Wheeler et al., 2019; Rojac et al., 2016; Son et al., 2020). Among them, metal oxide semiconductors are more valuable due to their convenience in the preparation process. However, their applications often face challenges such as complex preparation processes and insufficient stability. Cu_2_O has been extensively studied as a metal oxide photocathode due to its suitable bandgap and high carrier separation efficiency. Its severe photocorrosion makes it heavily reliant on a protective layer to ensure its stability (Li et al., 2019; Ying et al., 2018; Kim et al., 2019). Therefore, the quest for efficient and stable photocathode materials remains a priority.

Iron-based oxides are among the few photoelectrode materials that maintain excellent stability in PEC reactions. Particularly, hematite (α-Fe_2_O_3_) is one of the most abundant and low-cost semiconductor materials capable of absorbing visible light with a bandgap of around 2.1 eV. Although most Fe^3+^ containing binary and ternary oxides are n-type semiconductors, studies have shown that doping α-Fe_2_O_3_ with cations such as Mg^2+^, Zn^2+^, and Cu^2+^ or anions like N^3-^ can induce p-type semiconductor properties (Morikawa et al., 2011; Qi et al., 2013). Takeshi Morikawa’s group prepared N,Zn co-doped p-type α-Fe_2_O_3_ by magnetron co-sputtering and annealing. This N,Zn-Fe_2_O_3_ exhibited excellent photocurrent performance due to good light response and high charge carrier concentrations (Morikawa et al., 2013). Furthermore, transitioning semiconductors from n-type to p-type conductivity by altering doping concentrations has been reported in other semiconductor studies. Junpeng Wang’s group synthesized p-type tetragonal zirconia-phase BiVO_4_ films via a hydrothermal method, which demonstrated hydrogen production activity without any external bias due to their p-type conductivity and built-in electric fields in the liquid/semiconductor interface (Wang J. et al., 2019). Jiatao Zhang’s group employed a cation exchange-based doping method to convert n-type Au@CdS core-shell nanocrystals into p-type ones by doping Cu^+^ into CdS and constructed tandem PEC cells with undoped and Cu-doped Au@CdS photocathodes, achieving stable H_2_ and O_2_ evolution under light irradiation without external bias or cocatalysts (Pan et al., 2019).

However, researches on stable and cost-effective Fe-based oxide photocathode materials remain limited, especially on the one-step synthesis of p-type Fe_2_O_3_. In this work, we successfully prepared Fe_2_O_3_ spindle-like nanoarrays grown directly on FTO substrates through hydrothermal synthesis and subsequent heat treatment. These Fe_2_O_3_ nanoarrays exhibited p-type semiconductor conductivity characteristics and enabled PEC water splitting to produce hydrogen under illumination. The determination of their energy band structure provides valuable insights for future research on iron-based oxides as photocathodes.

## 2 Experimental section

### 2.1 Synthesis of p-type Fe₂O₃ spindle-like nanoarrays

The fluorine-doped tin oxide (FTO) substrates with sizes of 1.5 cm × 3.0 cm were cleaned in ultrasonic cleaner with acetone, anhydrous ethanol, and deionized water for 30 min, respectively. Then, they were dried for subsequent use. 0.2173 g potassium ferricyanide was dissolved in 10 mL deionized water with stirring to get the transparent solution. A piece of FTO was placed obliquely in a polytetrafluoroethylene (PTFE) liner with the conductive side facing down. Then, the potassium ferricyanide solution was added. After sealing the reactor, a hydrothermal reaction was conducted at 180°C for 6 h. When the reactor was allowed to cool naturally to room temperature, the FTO substrate was taken out, thoroughly rinsed with deionized water, and dried in an oven at 60°C. Finally, it was heat-treated in a muffle furnace at 650°C for 3 h in an air atmosphere to obtain Fe₂O₃ arrays, labeled as p-Fe₂O₃.

### 2.2 Synthesis of n-type Fe₂O₃ nanoarrays

The n-type Fe₂O₃ nanorod arrays were prepared as reference according to a method reported in the literature. The specific procedure was as follows (Lim et al., 2017): 1.9465 g anhydrous ferric chloride and 1.2132 g potassium nitrate were weighed and dissolved to 12 mL deionized water. The mixture was stirred until the solution became clear and transparent. Then, the pH value of the solution was adjusted to 1.5 by adding concentrated hydrochloric acid. The hydrothermal reaction was conducted at 95°C for 4 h with FTO as substrate. Afterwards, the FTO was rinsed with deionized water, dried in an oven at 60°C, and nanorod-like β-FeOOH arrays were obtained. The sample was then heat-treated in a muffle furnace in an air atmosphere at 800°C for 10 min and labeled as n-Fe₂O₃.

### 2.3 Characterization

The D8 Discover X-ray diffractometer from Bruker was utilized to investigate the phase composition of the samples. The surface element composition, valence states, and the estimation of the valence band spectrum position of the semiconductor material were accomplished using an X-ray photoelectron spectrometer (Thermo Fisher Scientific K-Alpha). The Hitachi S-4800 scanning electron microscope (SEM) and the Tecnai G2 field-emission transmission electron microscope (TEM) equipped with energy disperse spectroscopy (EDS) were employed to observe the microstructural morphology of the samples. Additionally, UV-visible diffuse reflectance tests were conducted using a Shimadzu UV-3600 UV-visible spectrophotometer.

### 2.4 Photoelectrochemical measurements

All electrochemical and photoelectrochemical performance in this work were conducted on a Zahner IM6 electrochemical workstation from Zennium. A 300 W Xe lamp equipped with an AM 1.5G filter (100 mW cm⁻^2^) was utilized to simulate solar illumination for the photoelectrochemical performance tests. A three-electrode system was employed, consisting of the prepared film electrode as the working electrode, a platinum sheet as the counter electrode, and a calomel electrode as the reference electrode, with 0.1 mol L⁻^1^ potassium hydroxide solution serving as the electrolyte. Linear sweep voltammetry (LSV) curves were recorded under chopped light conditions, with a light-switching interval of 3 s and a scan rate of 5 mV s⁻^1^. Amperometric (I-t) curves were obtained by applying a specific bias voltage to the working electrode and observing the resulting changes in current over time. Additionally, Mott-Schottky plots were measured in dark conditions at a frequency of 1,000 Hz.

## 3 Results and discussion

**FIGURE 1:**
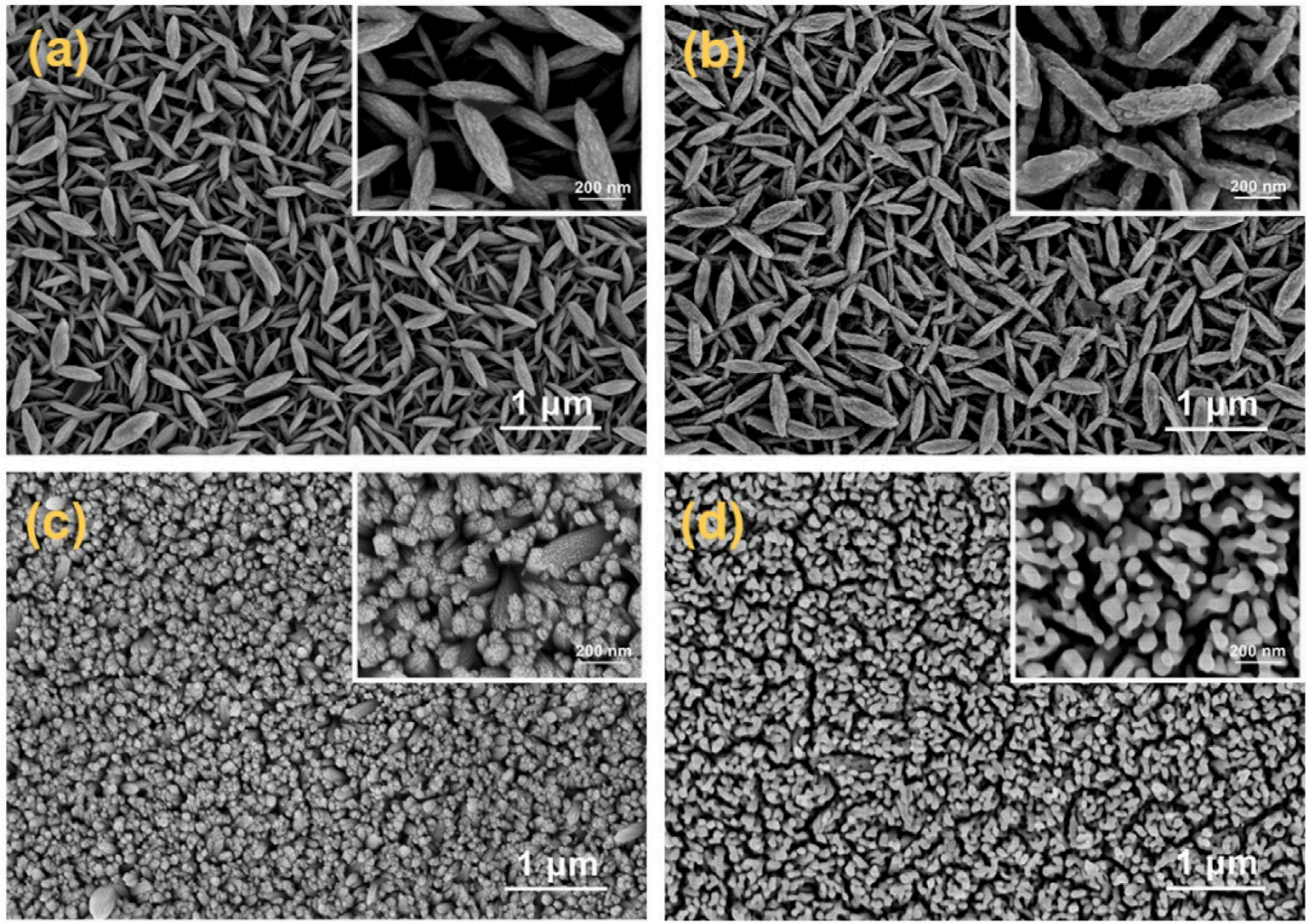
SEM images of p-Fe_2_O_3_ arrays **(A)** before heat treatment and **(B)** after heat treatment, SEM images of n-Fe_2_O_3_ nanorod arrays **(C)** before heat treatment and **(D)** after heat treatment (inset is the enlarged image).

The micromorphology and structural characteristics of the as-prepared p-Fe_2_O_3_ nanoarrays were observed through SEM and TEM images. As shown in Figure 1A, a spindle-like nanoarray morphology was obtained on the FTO substrate after the hydrothermal process. These spindles were uniformly arranged perpendicular to the substrate, with lengths ranging from 400 to 500 nm and thicknesses of 30–50 nm. After heat treatment at 650°C, as shown in Figure 1B, the surface of p-Fe₂O₃ became relatively rough, possibly due to the removal of CN⁻ groups originally coordinated with surface iron ions during high temperature heat treatment. Notably, the morphology and size of the p-Fe₂O₃ spindles did not change significantly. This perpendicular structure to the substrate facilitates the rapid transfer of photogenerated holes to the substrate in the vertical direction and the effective migration of photogenerated electrons to the electrode surface in the horizontal direction, thereby effectively promoting the separation of photogenerated carriers. Furthermore, the large specific surface area of p-Fe₂O₃ provides abundant active sites, conducive to efficient photoelectrochemical reactions at the interface between the photoelectrode and the electrolyte. Regarding the morphology of n-Fe₂O₃ (see Figures 1C, D), tightly packed vertical nanorod arrays were synthesized on an FTO substrate via hydrothermal methods, similar to what has been reported in the literature (Lim et al., 2017). After annealing, the nanorod arrays become relatively loose due to the removal of hydroxyl groups.

**FIGURE 2:**
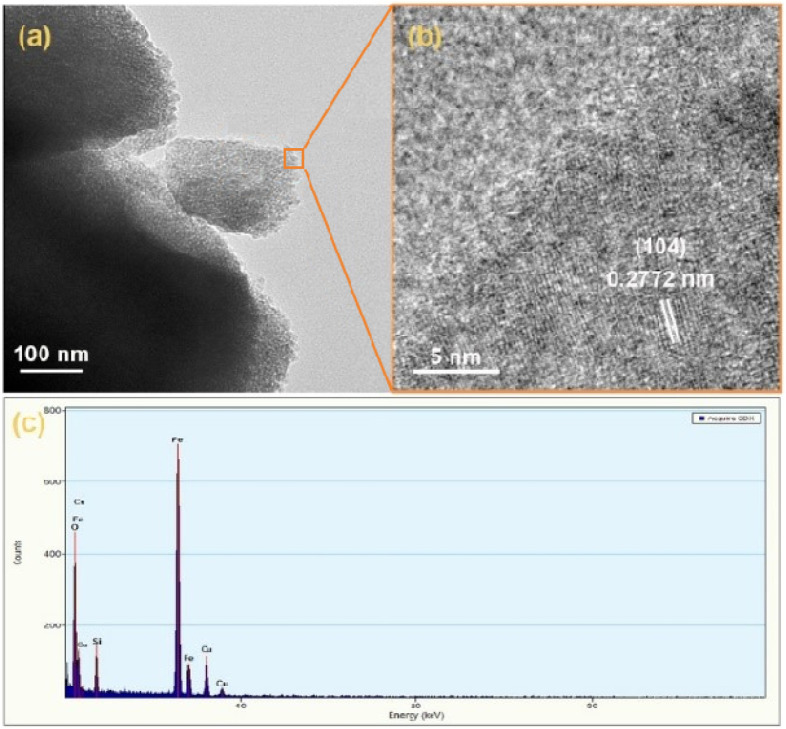
**(A)** TEM image, **(B)** HRTEM image and **(C)** EDS spectrum of p-Fe_2_O_3_.

Figure 2A shows the TEM image of p-Fe₂O₃, where the spindle-like structure of the obtained p-Fe₂O₃ was further confirmed. From the HRTEM image in Figure 2B, the lattice spacing of the p-Fe₂O₃ is measured as 0.2772 nm, corresponding to the (104) plane of α-Fe₂O₃. In the EDX spectrum shown in Figure 2C, apart from the Cu element from the Cu grid and the Si element from the glass substrate, only Fe and O elements are observed, preliminarily confirming that the p-Fe₂O₃ array is iron-based oxides.

**FIGURE 3:**
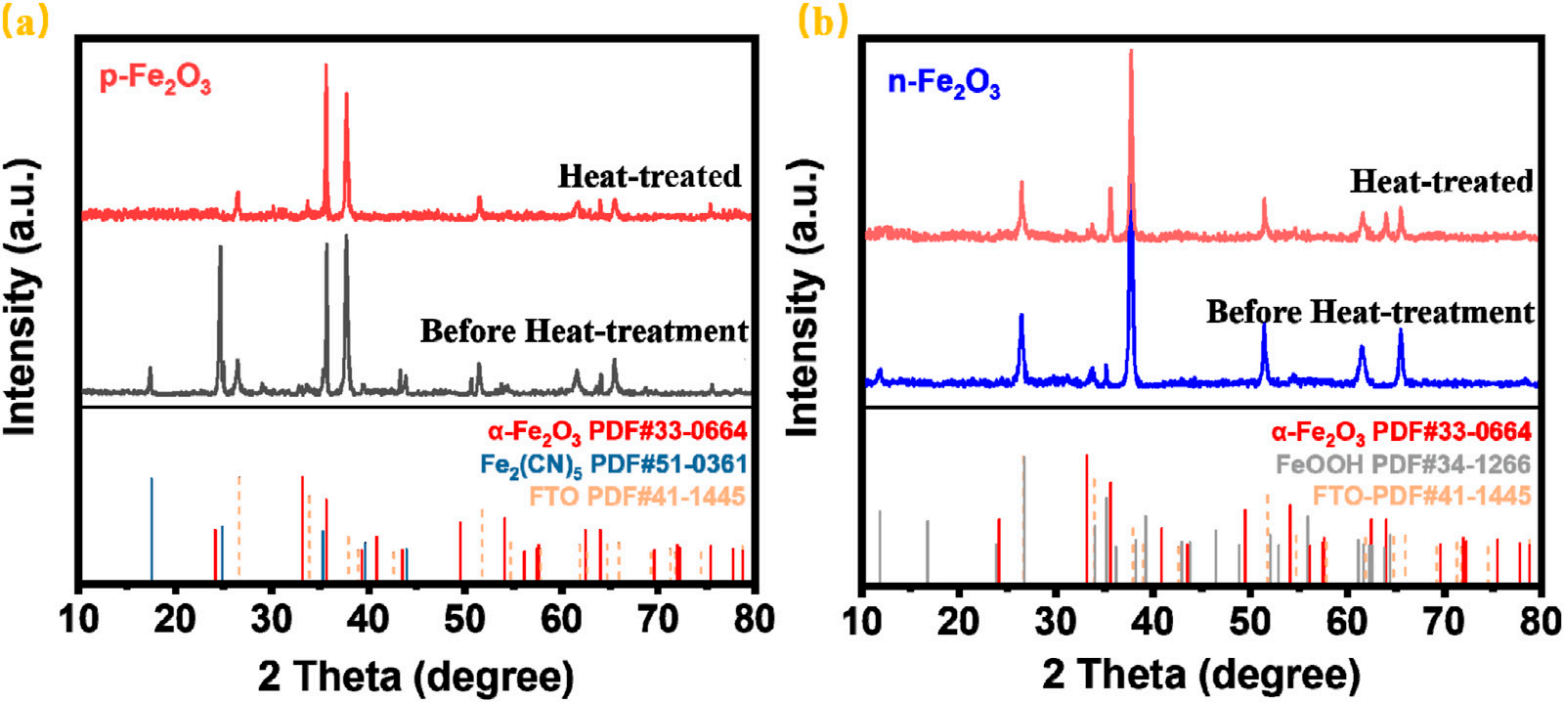
**(A)** XRD patterns of **(A)** p-Fe_2_O_3_ and **(B)** n-Fe_2_O_3_.

The phase composition of the Fe₂O₃ nanoarrays was characterized by X-Ray diffraction (XRD). Figure 3A shows the XRD patterns of the p-Fe₂O₃ nanoarrays before and after heat treatment. As shown, peaks at 26.6°, 33.8°, 37.8°, 51.7°, 61.7° and 65.7° correspond to the (110), (101), (200), (211), (310) and (301) planes SnO_2_ (JCPDS NO. 41–1,445) from the FTO substrate. The XRD pattern of the untreated p-Fe₂O₃ mainly shows two phases. The diffraction peak at 35.6°originates from the (110) plane of α-Fe₂O₃ (JCPDS NO. 33-0664). The diffraction peaks at 17.5° and 24.8° correspond to the (200) and (220) planes of Fe₂(CN)₅·H₂O (JCPDS NO. 51-0361), indicating coordination between iron ions and CN^−^ during hydrothermal synthesis. After heat treatment, only the crystallinity peaks at 33.2° and 35.6° can be verified in addition to the peaks corresponding to FTO, which are attributed to the (104) and (110) crystal planes of hematite (JCPDS 33-0664). The diffraction peaks of Fe₂(CN)₅·H₂O completely disappear, confirming the successful synthesis of the α-Fe_2_O_3_. Similarly, Figure 3B presents the XRD patterns of n-Fe_2_O_3_ nanoarrays before and after heat treatment. Apart from the FTO peaks, the untreated sample only shows peaks at 11.8° and 35.1°, corresponding to the (110) and (211) planes of β-FeOOH (JCPDS NO. 34-1266). After heat treatment, the diffraction peaks of β-FeOOH vanish, and new peaks at 33.2° and 35.6° appear, corresponding to the (104) and (110) planes of α-Fe_2_O_3_. Phase analysis indicated that both the prepared p-Fe₂O₃ and n-Fe₂O₃ arrays were α-Fe₂O₃.

**FIGURE 4:**
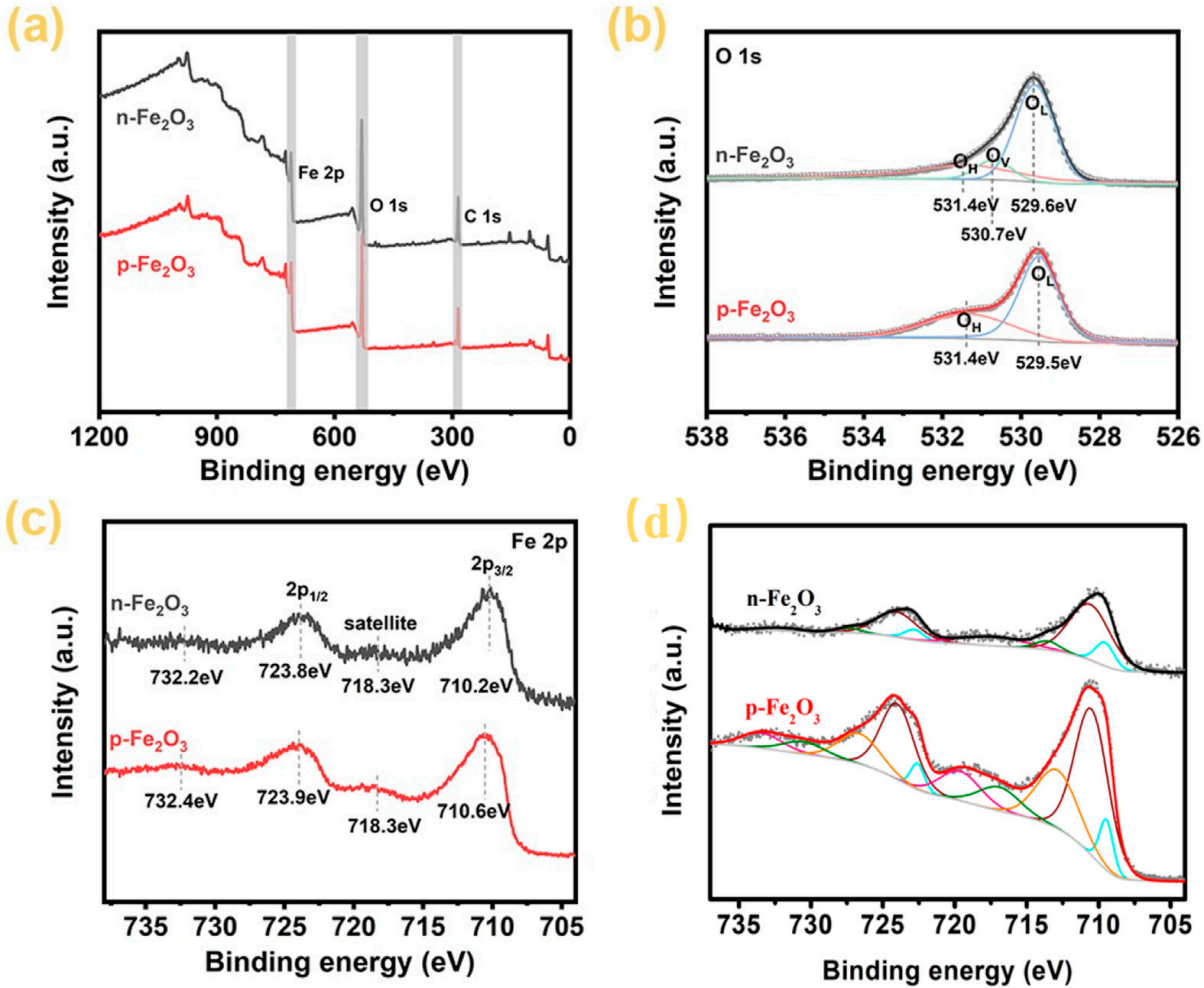
XPS spectra of Fe_2_O_3_ arrays, **(A)** survey spectrum, **(B)** high-resolution O 1s spectrum, **(C)** high-resolution Fe 2p spectrum, **(D)** peak fitting of Fe 2p spectrum.

To further analyze the elemental chemical composition and valence states of the Fe_2_O_3_ nanoarrays, X-ray photoelectron spectroscopy (XPS) tests were conducted. The full spectra in Figure 4A confirm that both p-Fe₂O₃ and n-Fe₂O₃ contain only Fe and O elements, consist with XRD results. Figure 4B shows the high-resolution spectrum of O 1s. The O 1s orbital of p-Fe₂O₃ can be fitted into two peaks located at 529.5 and 531.4 eV, corresponding to lattice oxygen (O_L_) and surface chemisorbed oxygen or water molecules (O_H_), respectively. The O 1s orbital of n-Fe₂O₃ can be fitted into three peaks at 529.6, 530.7, and 531.4 eV, corresponding to lattice oxygen (O_L_), oxygen vacancies (O_V_) and surface chemisorbed oxygen or water molecules (O_H_), respectively (Qin et al., 2023). The presence of oxygen vacancies is the reason for the n-type conductivity of n-Fe₂O₃ (Qi et al., 2013). The high-resolution spectra of Fe 2p reveal differences in iron valence states between p-Fe₂O₃ and n-Fe₂O₃. As shown in Figure 4C, the Fe 2p_3/2_ orbital of the n-Fe₂O₃ is located at 710.2 eV, with a corresponding satellite peak at 718.3 eV and the Fe 2p_1/2_ orbital is located at 723.8 eV, with a corresponding satellite peak at 732.2 eV. These peak positions match well with compound containing Fe predominantly in the form of Fe^3+^ (Wang et al., 2022). For the p-Fe₂O₃, similar plotting could be observed, with the Fe 2p_3/2_ orbital peak shifts towards slightly higher energy compared to that of n-Fe₂O₃, located at 710.6 eV. This is due to the generation of excess holes around iron ions to achieve charge balance in the system. For a detailed analysis of the iron valence states, peak fittings of the main Fe 2p spectrum are presented in Figure 4D, where the experimental Fe 2p, Fe^4+^, Fe^3+^, Fe^2+^, background and simulated plots are gray dotted, orange, wine, blue, gray and black/red lines, respectively. The olive and pink plots represent the satellite of 2p_1/2_ and Fe 2p_3/2_, respectively. The results indicate that Fe in n-Fe₂O₃ exists in the form of Fe^3+^ and Fe^2+^, while Fe in p-Fe₂O₃ is deconvoluted into three components: Fe^4+^, Fe^3+^, and Fe^2+^ (Wheeler et al., 2019; Li et al., 2021). The presence of Fe^4+^ in p-Fe₂O₃ is attributed to the localization of extra holes around Fe^3+^ ions, which is might due to intrinsic defects such as metal vacancies or substitution of Fe^3+^ by Fe^2+^ during the phase transition via high-temperature annealing process. This is the fundamental reason for the p-type conductivity semiconductors (Rojac et al., 2016; Lumley et al., 2019).

**FIGURE 5:**
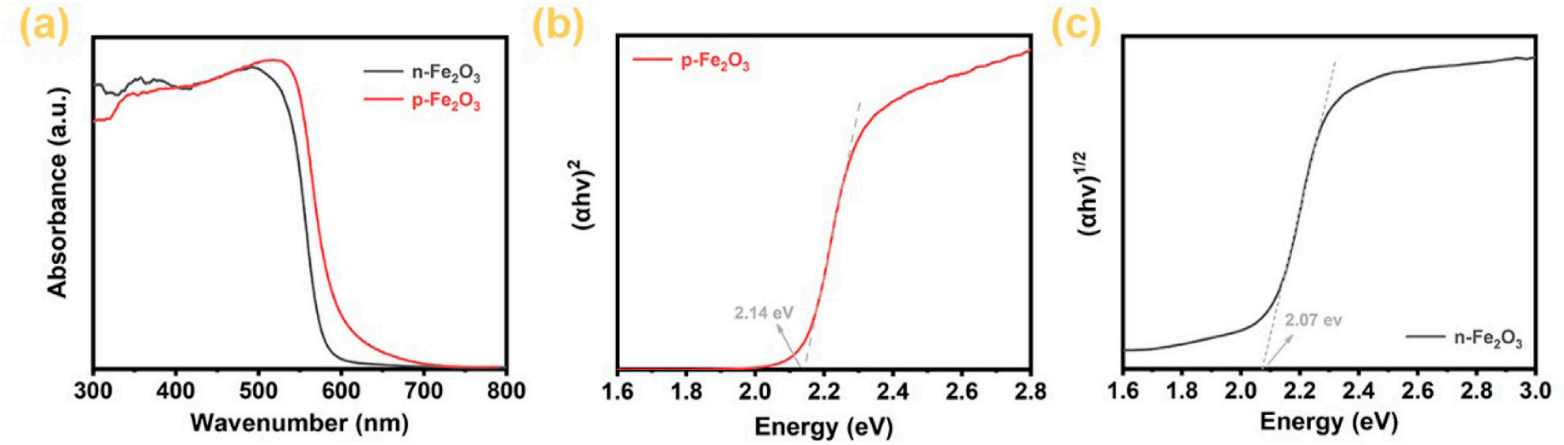
**(A)** UV-vis absorption spectra of Fe_2_O_3_ arrays, Tauc plots of **(B)** p-Fe_2_O_3_ and **(C)** n-Fe_2_O_3_.

Figure 5A presents the UV-visible absorption spectra of p-Fe₂O₃ and n-Fe₂O₃. It is clearly observed that both oxides can absorb all UV light and a significant portion of visible light. Specifically, the maximum absorption edge of n-Fe₂O₃ is located at 590 nm, while that of p-Fe₂O₃ is slightly higher, reaching 600 nm. By converting the UV-visible absorption spectra into Tauc plot curves and extending the tangent line of the linear portions to intersect the *x*-axis, the bandgap of the semiconductor can be obtained. From Figure 5B, the bandgap of the p-Fe₂O₃ nanoarray is 2.14 eV, whereas Figure 5C indicates that the bandgap of the n-Fe₂O₃ nanorod array is 2.07 eV. The narrow bandgap of Fe₂O₃ enables it to absorb sufficient visible light, providing a significant advantage for its application in photoelectrochemical water splitting.

**FIGURE 6:**
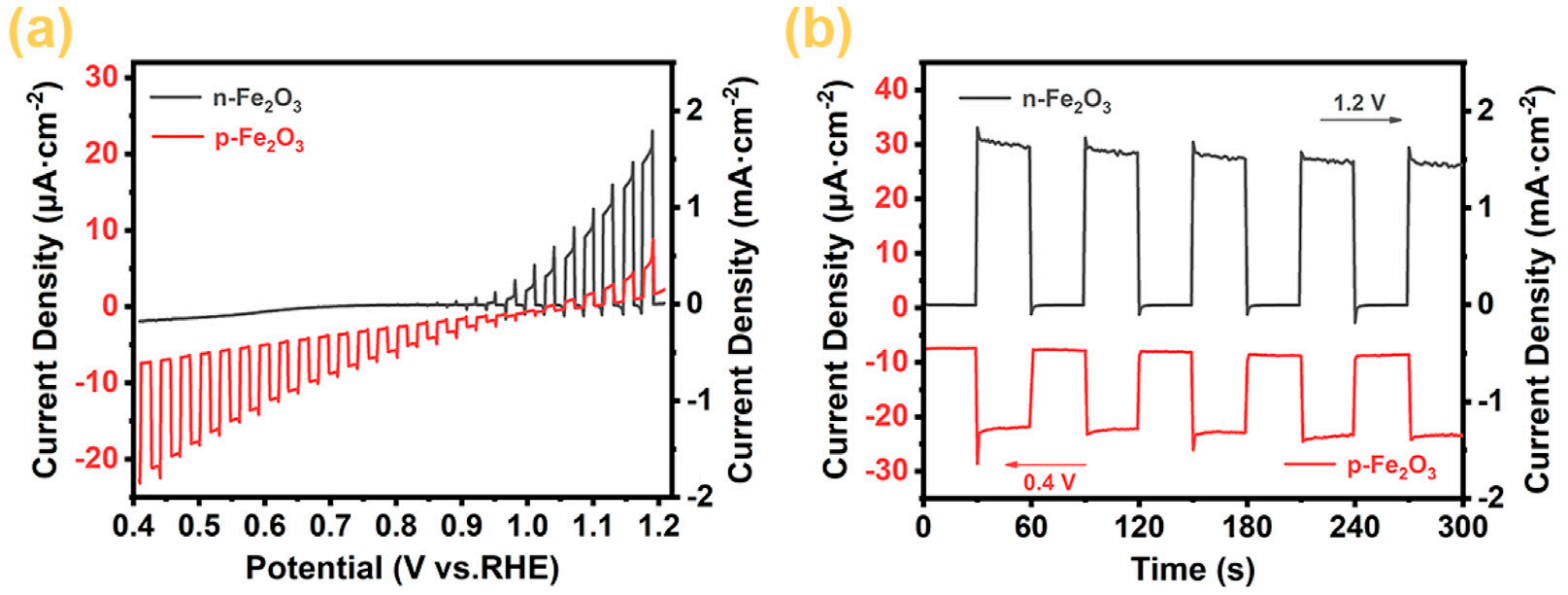
**(A)** Linear sweep voltammograms (LSV), **(B)** amperometric i–t curves under chopped light illumination of Fe_2_O_3_ arrays.

The photoelectrochemical water splitting performance of p-Fe₂O₃ spindle-shaped nanoarrays and n-Fe₂O₃ nanorod arrays was investigated through photoelectrochemical measures. Figure 6A shows the linear scan voltammetry curves under chopped light. As reported in literature, forward scanning was used to characterize the photoelectrochemical oxidation performance of n-Fe₂O₃. As the voltage increases, the anodic photocurrent also increases, with an onset potential of approximately 0.9 V (vs RHE) and a photocurrent reaching 1.70 mA cm⁻^2^ at 1.23 V (vs RHE). To characterize the reduction performance of p-Fe₂O₃, reverse scanning was employed. When the voltage decreased to 1.0 V, the current began to increase in the negative direction, indicating the electrode’s photocathode characteristics and confirming that the prepared p-Fe₂O₃ spindle-shaped nanoarrays is a p-type semiconductor. The cathodic photocurrent reaches −23.5 μA cm^−2^ at 0.4 V (vs. RHE).

Figure 6B shows the chronoamperometry curves under chopped light. Both Fe₂O₃ materials exhibit rapid light response, indicating that these materials have prompt transfer ability of photogenerated electrons and holes. Specifically, n-Fe₂O₃ achieves an anodic photocurrent response of 1.5 mA cm^−2^ with anodic overpotential at 1.2 V (vs RHE), while p-Fe₂O₃ exhibits a stable cathodic photocurrent of −23 μA cm^−2^ with cathodic overpotential at 0.4 V (vs RHE). The electrochemical test results demonstrate that the prepared n-Fe₂O₃ is an n-type semiconductor suitable for PEC water oxidation reactions, while the prepared p-Fe₂O₃ is a p-type semiconductor with PEC catalytic water reduction performance.

**FIGURE 7:**
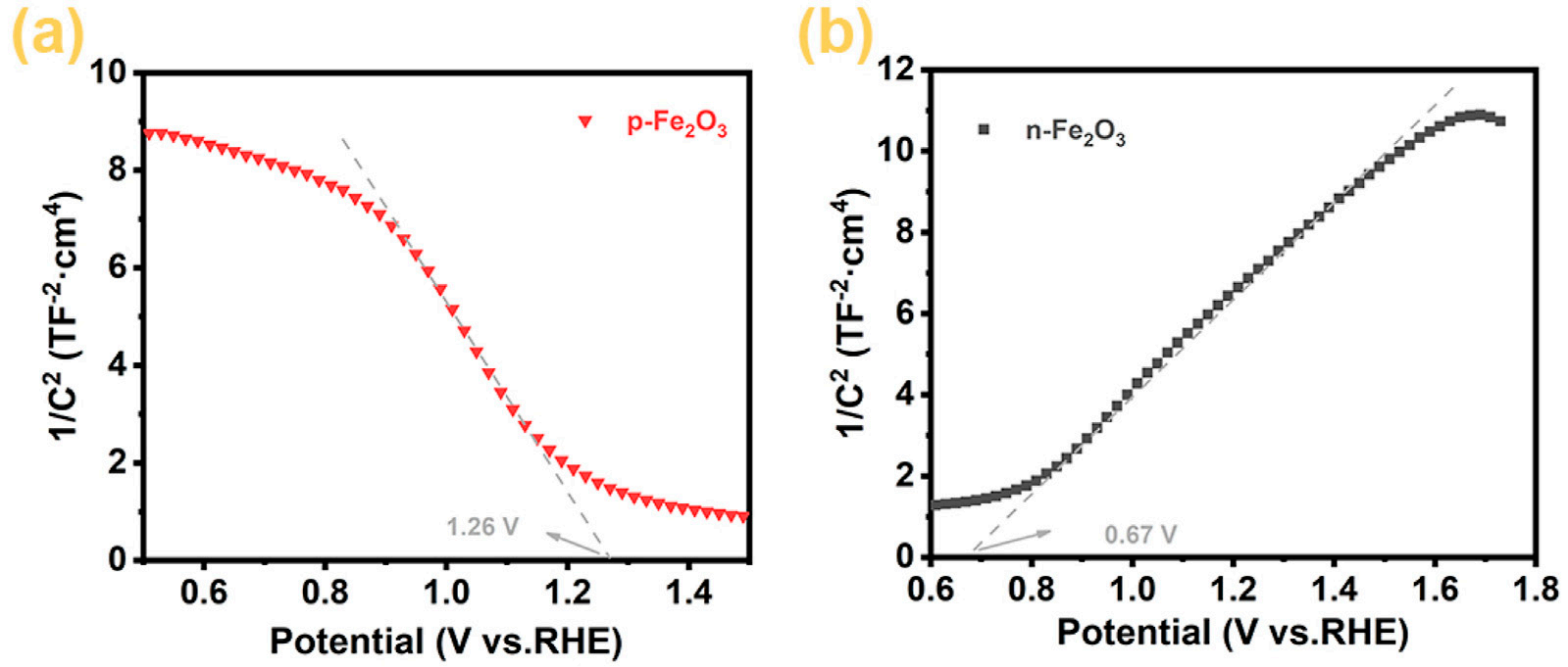
Mott-Schottky curves of **(A)** p-Fe_2_O_3_ and **(B)** n-Fe_2_O_3_.

To more conclusively verify the semiconductor types of the Fe₂O₃ nanoarrays, we measured the Mott-Schottky curves of the p-Fe₂O₃ spindle-shaped nanoarray and n-Fe₂O₃ nanorod array, with results shown in Figure 7. The negative slope of the p-Fe₂O₃ curve indicates that the prepared p-Fe₂O₃ spindle-shaped nanoarray is a p-type semiconductor, consistent with the results in the electrochemical tests. Meanwhile, the curve for n-Fe₂O₃ exhibits a positive slope, verifying that the n-Fe₂O₃ is a n-type semiconductor. According to the principles of Mott-Schottky curves, the flat band potential of the semiconductor can be estimated by extending the tangent line of their linear portions to the potential axis. From Figure 7A, the flat band potential of p-Fe₂O₃ is approximately 1.26 V (vs. RHE). Similarly, from the Mott-Schottky curve of the n-Fe₂O₃ nanorod array in Figure 7B, it can be inferred that n-Fe₂O₃ is an n-type semiconductor with a flat band potential of approximately 0.67 V (vs. RHE).

**FIGURE 8:**
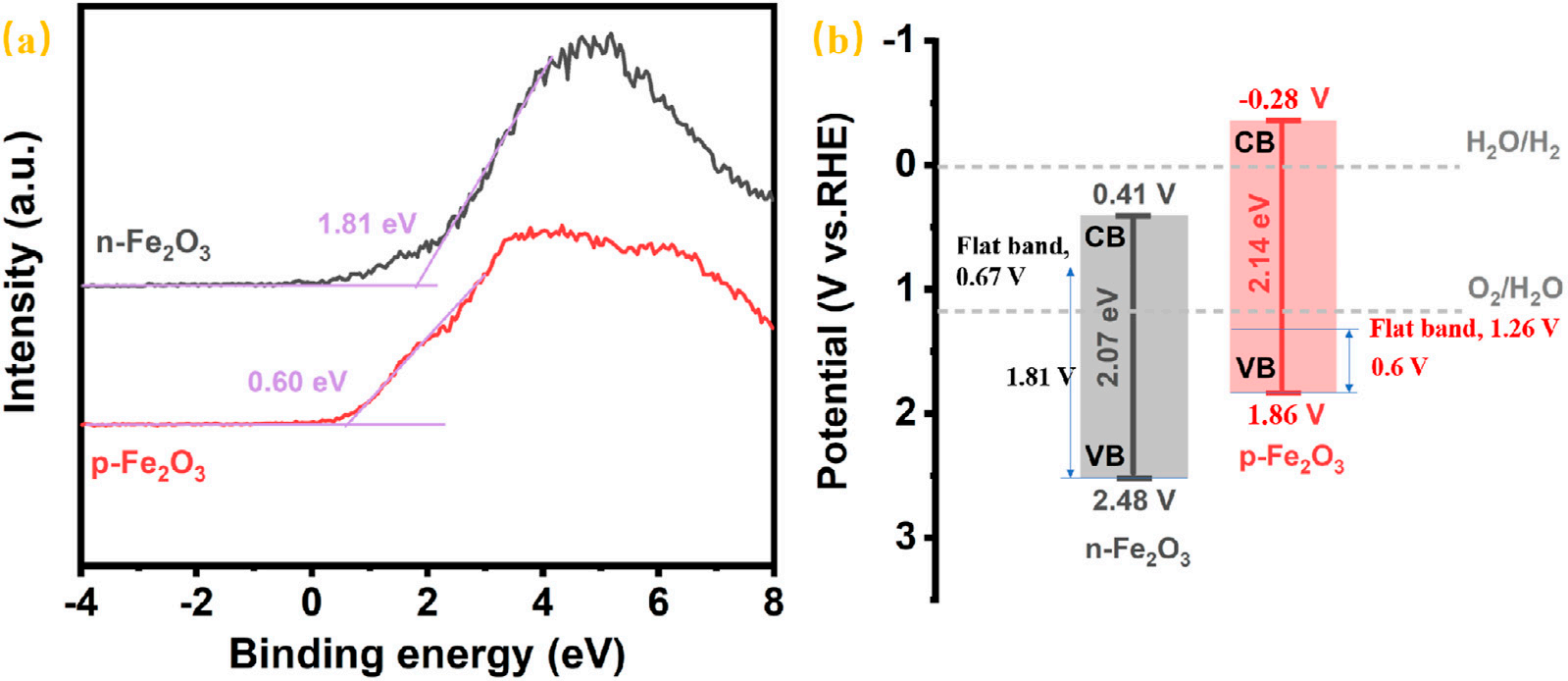
**(A)** The XPS valence band spectra and **(B)** schematic illustration of the band alignment of p-Fe_2_O_3_ and n-Fe_2_O_3_.

Since the reports on α-Fe₂O₃ with p-type semiconductor conductivity characteristic used as photocathode are still limited, further analysis of the conduction and valence band positions of the as-prepared p-Fe₂O₃ was conducted to provide reference for future research on iron-based oxides as p-type semiconductor. Figure 8A presents the valence band spectra measured by XPS, where the distance between the valence band edge and the Fermi level in a semiconductor can be determined by the intersection of the tangent to the curve and the horizontal line. From the figure, the distance between the valence band edge and the Fermi level of n-Fe₂O₃ is 1.81 eV, while that of p-Fe₂O₃ is 0.60 eV. Combined the flat band potentials obtained from the Mott-Schottky curves in Figure 7, the valence band edge can be estimated by summing the band potential and interval of the valence band edge and the Fermi level. Furthermore, together with the bandgap from the Tauc plots in Figure 5, the conduction band can be calculated. Thus, the prepared p-Fe₂O₃ is located at 1.86 V (vs. RHE) and the conduction band edge at −0.28 V (vs. RHE). For n-Fe₂O₃, the valence band edge and conduction band edge are located at 2.48 V (vs RHE) and 0.41 V (vs. RHE), respectively.

The corresponding energy band structure diagrams of the p-Fe₂O₃ and n-Fe₂O₃ nanoarrays are illustrated in Figure 8B. It can be seen that both Fe₂O₃ possess narrow bandgap, where p-Fe₂O₃ has a slightly wider bandgap than n-Fe₂O₃. The conduction band edge of n-Fe₂O₃ is higher than the hydrogen evolution potential, which is unfavorable for hydrogen evolution reactions. In contrast, the conduction band edge of p-Fe₂O₃ is below the hydrogen evolution potential, indicating that p-Fe₂O₃ can provide a certain photovoltage during the hydrogen evolution reaction, thereby effectively mitigating the slow water reduction kinetics. Therefore, p-Fe₂O₃ is a promising photocathode for catalyzing hydrogen evolution.

## 4 Conclusion

In the present study, Fe₂O₃ spindle-shaped nanoarrays on FTO substrates with p-type semiconducting properties were successfully synthesized *via* a hydrothermal method coupled with subsequent heat treatment. Under illumination, these arrays exhibited a cathodic photocurrent density of −23 μA cm^-2^ at 0.4 V (vs. RHE). Further investigation of their energy band structure revealed that the conduction band edge is positioned below the hydrogen evolution potential, favoring the hydrogen evolution reaction. As an abundant and low-cost material on earth, Fe_2_O_3_ has great potential as a p-type semiconductor. This study provides a reference for subsequent research on p-type semiconductors and their applications in water splitting.

## Data Availability

The original contributions presented in the study are included in the article/supplementary material, further inquiries can be directed to the corresponding authors.
